# Anti-Cholinesterase Activity of *Lycopodium* Alkaloids from Vietnamese *Huperzia squarrosa* (Forst.) Trevis

**DOI:** 10.3390/molecules191119172

**Published:** 2014-11-19

**Authors:** Nguyen Ngoc Chuong, Nguyen Thi Thu Huong, Tran Manh Hung, Tran Cong Luan

**Affiliations:** 1 Research Center of Ginseng and Medicinal Materials, 41 Dinh Tien Hoang, District 1, Ho Chi Minh City 700-00, Vietnam; 2 Faculty of Chemistry, University of Science, Vietnam National University-HoChiMinh City, 227 Nguyen Van Cu, District 5, Ho Chi Minh City 227-01, Vietnam

**Keywords:** *Huperzia squarrosa*, Lycopodiaceae, lycosquarosine A, acetylcholinesterase, *Lycopodium* alkaloids

## Abstract

A series of *Lycopodium* alkaloids, namely lycosquarosine A (**1**), acetylaposerratinine (**2**), huperzine A (**3**), huperzine B (**4**), 8α-hydrophlemariurine B (**5**), and huperzinine (**6**), has been isolated from Vietnamese *Huperzia squarrosa*. Among them, lycosquarosine A (**1**) is the new metabolite of the natural source. Lycosquarosine A completely inhibited AChE activity in a dose dependent manner with an IC_50_ value of 54.3 μg/mL, while acetylaposerratinine (**2**) showed stronger inhibitory activity than **1** with an IC_50_ value of 15.2 µg/mL. This result indicates that these alkaloids may be a potent source of AChE inhibitors.

## 1. Introduction

Alzheimer’s disease (AD) is a neurodegenerative disease and the most frequent and predominant cause of dementia among the elderly, provoking progressive cognitive decline, psychobehavioral disturbances, memory loss, presence of senile plaques, neurofibrillary tangles and a decrease in cholinergic transmission [1,2]. Neuropathological evidence has demonstrated that cholinergic functions decline in the basal forebrain and cortex in senile dementia of the Alzheimer type [3]. Accordingly, the enhancement of cholinergic neurotransmission has been considered as one potential therapeutic approach against AD. Although the pathogenesis of AD is complicated and involves numerous pathways, two major hypotheses are currently under consideration regarding the molecular mechanism: the cholinergic hypothesis and the amyloid cascade hypothesis. Thus, the focus herein is upon selective cholinesterase (ChE) inhibitors in order to alleviate cholinergic deficits and improve neurotransmission. Pursuant to this, both could be established as viable therapeutic targets for AD [3,4,5]. One treatment strategy to enhance the cholinergic function is the use of acetylcholinesterase (AChE, EC 3.1.1.7) inhibitors to increase the amount of acetylcholine, which is present in the synapses between cholinergic neurons [6]. AChE inhibitors such as donepezil, rivastigmine and galantamine, which are the most extensively studied AChE inhibitors, have been shown to significantly improve cognitive function in AD [7,8].

Club moss (Lycopodiaceae) species are well-known to be a rich source of *Lycopodium* alkaloids possessing a complex heterocyclic ring system and wide ranging biological properties that have attracted great interest from biogenetic, synthetic, and biological perspectives [9,10,11]. Huperzine A, a famous C_16_N_2_-type alkaloid isolated from the Chinese folk medicinal herb *Huperzia serrata*, has been shown to be a highly potent, specific, and reversible inhibitor of AChE [10,12]. Until now, more than 300 *Lycopodium* alkaloids were reported [9,10,11]. Most of the *Lycopodium* alkaloids possessing AChE inhibitory activity such as huperzine A, huperzine B, and *N*-methylhuperzine B belong to the lycodine class [12,13]. In our continuing efforts to search for structurally interesting and bioactive *Lycopodium* alkaloids, especially in *Lycopodium* spp. from Vietnam, a new C_16_N_1_-type alkaloid, lycosquarosine A was isolated together with five known *Lycopodium* alkaloids from the club moss *Huperzia squarrosa* (Forst.) Trevis. Previously, *Lycopodium squarrosum* (*H. squarrosa*) originally from Thailand, was phytochemically investigated and several fawcettimine related alkaloids were described [14]. In this paper, we describe the isolation and structure elucidation of lycosquarosine A (**1**) and the other *Lycopodium* alkaloids **2**–**6** as well as their anti-cholinesterase activity.

## 2. Result and Discussion

**Figure 1:**
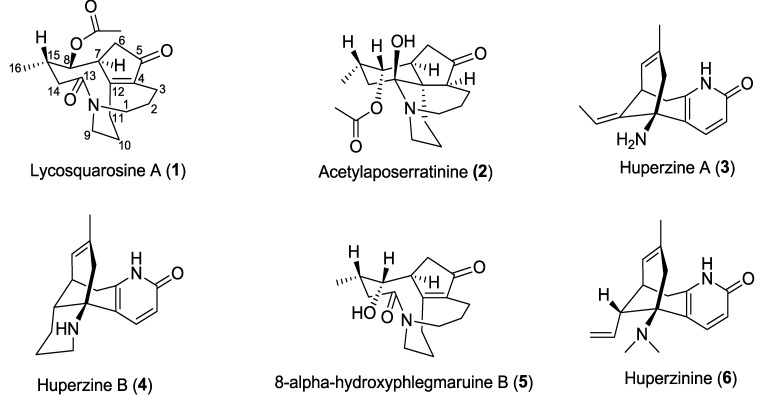
Chemical structures of isolated compounds **1**–**6**.

The MeOH extract of the club moss *H. squarrosa* was partitioned into *n*-hexane-, EtOAc-, and *n*-BuOH-soluble fractions and a H_2_O layer. Chromatographic purification of the EtOAc-soluble fraction led to the isolation of six compounds **1**–**6** (Figure 1).

**Figure 2:**
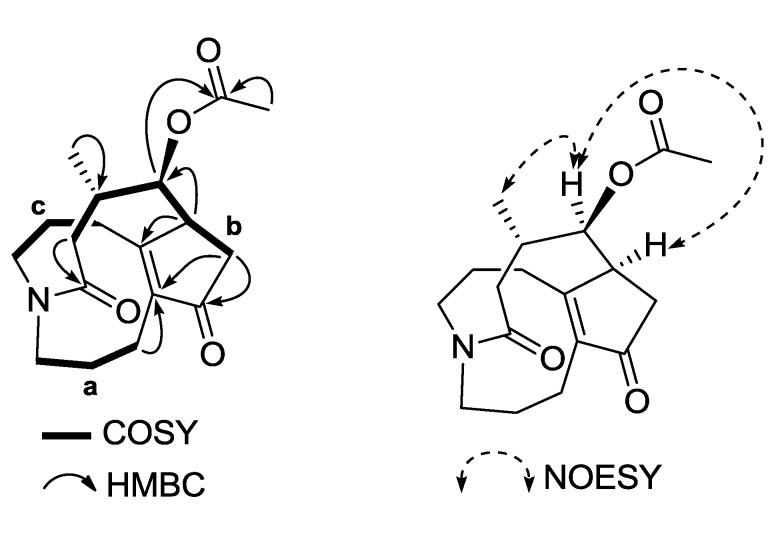
Selected 2D NMR correlations of **1**.

Lycosquarosine A (**1**) was obtained as a colourless amorphous solid and its molecular formula was deduced from HR-EI-MS analysis to be C_18_H_25_NO_4_. IR absorptions indicated the presence of a carboxylate functionality (1582 cm^−1^). Its ^13^C-NMR and DEPT spectra displayed signals for one methyl at δ_C_ 22.9 (C-16), two N-bearing methylenes at δ_C_ 50.6 (C-1 and C-9, two peak in overlap), six high-field methylenes at δ_C_ 18.4 (C-2), 22.1 (C-3), 37.4 (C-6), 25.1 (C-10), 30.3 (C-11) and 32.7 (C-14), one oxygenated methine at δ_C_ 79.6 (C-8), two methines at δ_C_ 45.0 (C-7) and 29.6 (C-15), together with four sp^2^ quaternary carbons at δ_C_ 205.7 (C-5), 173.1 (C-13), 169.8 (C-12) and 142.7 (C-4), indicating a phlegmariurine B-type related framework [15]. In addition, the carbon signals of one carbonyl δ_C_169.7 (C-17) and one methyl carbon at δ_C_ 20.9 (C-18) were ascribed to an acetoxyl group. The ^1^H-NMR spectrum of **1** displayed signals for a tertiary methyl of the acetoxyl group at δ_H_ 2.19 (3H, *s*, H-18), a secondary methyl at δ_H_ 1.08 (3H, *d*, *J* = 6.3 Hz, H-16), and one oxymethine proton at δ_H_ 5.06 (1H, *brd*, *J =* 5.0 Hz, H-8) (Table 1). By comparison with literature ^1^H- and ^13^C-NMR data [15,16], **1** could be assigned a phlegmariurine B carbon skeleton with a rearranged five member ring of a >C12=C4-C5(C=O)-C6-C7 type (Figure 1). The complete NMR assignments and connectivity of **1** were further determined by analysis of the COSY, HMQC and HMBC spectroscopic data. ^1^H–^1^H COSY and HSQC analyses indicated the presence of three carbon chains between H-1/H-2/H-3 (**a**), H-6/H-7/H-8/H-15/H-14 (**b**), and H-9/H-10/H-11 (**c**) shown by the bold lines in Figure 2. The long-range ^1^H–^13^C coupling (HMBC) observed between oxygenated methine H-8 and carbonyl carbon at δ_C_ 169.7 (C-17) confirmed the position of the acetoxyl group to be at C-8 (Figure 2 and Supplementary data). The ROESY correlation between H-16 and H-7 indicated that 1 had an α-oriented methyl group at C-15, which was similar to phlegmariurine type of *Lycopodium* alkaloids [15]. Furthermore, the β-orientation of the acetoxyl function located at C-8 was deduced from the ROESY experiment, showing ROE correlations between H_α_-7/H-16 and H-8. Thus, compound **1** was proved to be an 8β-acetoxyl derivative of phlegmariurine B, and was named lycosquarosine A

**Table 1:** ^1^H- (500 MHz) and ^13^C- (125 MHz) NMR data of lycosquarosine A (**1**).

Position	1 ^a^		
	δ_H_ ( *J* in Hz) ^b^		δ_C_
1	4.06 (1H, dd, 13.6, 3.6), 2.90 (1H, dt, 13.6, 3.0)		50.6
2	2.39 (1H, m), 1.41 (1H, m)		18.4
3	2.53 (1H, m), 2.46 (1H, m)		22.1
4			142.7
5			205.7
6	2.55 (1H, m), 2.03 (1H, brd, 19.0)		37.4
7	3.04 (1H, m)		45.0
8	5.06 (brd, 5.0)		79.6
9	3.96 (1H, td, 15.0, 3.0), 3.23 (1H, brd, 15.0)	50.6	
10	2.78 (1H, m), 1.93 (1H, m)	25.1	
11	2.98 (1H, m), 2.78 (1H, m)	30.3	
12		169.8	
13		173.1	
14	1.59 (1H, d, 15.5), 3.06 (1H, dd, 8.5, 15.5)	32.7	
15	2.56 (1H, m)	29.6	
16	1.08 (3H, d, 6.3)	22.9	
17		169.7	
18	2.19 (3H, s)	20.9	

^a^ Measured in mixture of MeOD and CDCl_3_; ^b^ Chemical shift may be overlapped signals which were confirmed by DEPT-135, HMQC, and HMBC experiments.

Compound **2** showed a pseudo-molecular ion peak at *m*/*z* 322 [M+H]^+^ in the ESI-MS, and the molecular formula, C_18_H_27_NO_4_, was established by HR-ESI-MS *m*/*z* 322.2051, [M+H]^+^. IR absorptions (1585 cm^−1^) implied the presence of a carboxylate functionality. ^1^H-, ^13^C-NMR and DEPT data revealed eighteen carbon signals due to four sp^2^ quaternary carbons, four sp^3^ methines, eight sp^3^ methylenes, and two methyl groups. Among them, two methylenes [(δ_C_ 54.9, δ_H_ 3.90 and 2.99) and (δ_C_ 50.9, δ_H_ 3.58 and 3.27), belonging to C-1 and C-9, respectively] were ascribed to those bearing a nitrogen. The ^1^H-NMR spectrum of **2** displayed signals for a tertiary methyl of the acetoxyl group at δ_H_ 2.14 (3H, *s*, H-18), a secondary methyl at δ_H_ 1.02 (3H, *d*, *J* = 6.5 Hz, H-16) which are similar with those of positions in **1**. Since no IR bands and ^13^C-NMR signals indicated a double bond in comparison with **1**, compound **2** must be pentacyclic, which suggested the possibility that a new ring was formed. Extensive NMR analyses spectra of **2** resembled those of **1** except for the presence of one carbinolamine moiety at δ_C_ 94.5 (C-13), a sp^2^ quaternary carbon at δ_C_ 47.5 (C-12) and a sp^3^ methine at C-4 position (δ_C_ 47.6, δ_H_ 2.40) instead of three quaternary carbons at the same positions in **1** (Table 1). Combination of HMQC and ^1^H–^1^H COSY also indicated the presence of three fragment carbon chains (**a**) –CH_2_CH_2_CH_2_CH–(C-1–C-4), (**b**) –CH_2_CHCHCHCH_2_– (C-6–C-8–C-15–C-14) and (**c**) –CH_2_CH_2_CH_2_–(C-9–C-11) (Figure 2). The long-range ^1^H-^13^C coupling (HMBC) observed between oxygenated methine H-8 and carbonyl carbon at δ_C_ 170.4 (C-17) confirmed the position of the acetoxyl group to be at C-8. The coupling pattern of the oxymethine resonance at δ_H_ 5.04 (1H, *brs*, H-8) and its oxygenated carbon at δ_C_ 72.3 (C-8) differed from that in **1** with δ_H_ 5.08 (1H, *brd*, *J =* 5.0 Hz) and δ_C_ 80.8, indicating for the α-orientation of the acetoxyl group which is in accordance with the orientation of phlegmariurine B [14]. Additionally, the relative stereochemistry of **2** was elucidated from NOESY correlations. From that, the α-orientation of the acetoxyl function was confirmed from the enhancement of the signals for H-6β methylene hydrogen and H-15 by H-8 irradiation. Other key NOESY correlations were observed between H-4 and H-7, and H_3_-16 indicating that they are on the same α-orientation. Thus, the relative stereochemistry of **2** was assigned. This compound named acetylaposerratinine [14]. 

The other compounds were identified as huperzine A (**3**), huperzine B (**4**) [12,13], 8α-hydrophlemariurine B (**5**) [15], and huperzinine (**6**) [13] by comparing their physiochemical and spectroscopic data with those reported in the corresponding literature.

AChE inhibitors increase the availability of acetylcholine in central cholinergic synapses and are currently the most promising available drugs for the treatment of Alzheimer’s disease [17]. Cholinergic interneurons in the striatum are an even richer source of acetylcholinesterase and would also be affected strongly by such enzyme inhibitors [17,18]. The anti-cholinesterase activity of the isolated alkaloids was tested by the Ellman reaction [19]. Because the known compounds **3**–**6** were already reported to have cholineseterase inhibitory activity, in this experiment, we tested only compounds **1** and **2** for AChE and BuChE inhibition. The new compound, lycosquarosine A (**1**) showed potent AChE inhibitory activity with an IC_50_ value of 54.3 µg/mL. However, acetylaposerratinine (**2**) showed stronger inhibitory activity than **1**, with an IC_50_ value of 15.2 µg/mL. Both of them exhibited weak inhibitory effects on BuChE, with IC_50_ values over 100 µg/mL. The results showed that lycosquarosine A (**1**) and acetylaposerratinine (**2**) exhibited selective inhibition for AChE compared with BuChE. Berberine, which was used as positive control [20], exhibited AChE and BuChE inhibitory activity with IC_50_ values of 0.09 and 8.01 µg/mL, respectively. 

## 3. Experimental Section

### 3.1. General Procedures

Optical rotations were measured with a DIP 370 digital polarimeter (JASCO, Tokyo, Japan). UV spectra were taken in MeOH using an Evolution^TM^ 300 Thermo Spectrometer (Thermo Fisher Scientific Inc., Waltham, MA, USA). The NMR spectra were obtained on a Unity Inova 500 MHz spectrometer (Varian, McKinley Scientific Inc., Sparta Township, NJ, USA). Silica gel (63–200 μm particle size, Merck, Seoul, Korea) and RP-18 (75 μm particle size, Merck) were used for column chromatography. TLC was carried out using Merck silica gel 60 F_254_ and RP-18 F_254_ plates. HPLC was carried out using a Waters (Waters Corporation, Milford, MA, USA) system (515 pump) equipped with a UV detector (486 Tunable Absorbance) and an YMC Pak ODS-A column (20 × 250 mm, 5 μm particle size, YMC Co., Ltd., Kyoto, Japan) and HPLC solvents were purchased from SK Chemicals, Seoul, Korea.

### 3.2. Plant Material

The *H. squarrosa* club moss was collected in Lam Dong Province, in the central area of Viet Nam on May 2012, and identified by Professor Luan TC, Department of Oriental Medicine, Ho Chi Minh City University of Medicine and Pharmacy. A voucher specimen (TCL 00116) was deposited at the Herbarium of the Research Center of Ginseng and Medicinal Materials, Ho Chi Minh City, Vietnam. 

### 3.3. Extraction and Isolation

The dried sample (2.5 kg) was extracted with MeOH (5 L) by refluxing three times for. The combined extracts were concentrated under reduced pressure to give the crude extract (502 g), which was then suspended in 5% HCl and partitioned with CH_2_Cl_2_. The aqueous layer was alkalinized until pH~10 with aqueous ammonia, preparing for submitting to Diaion HP 20 macroporous resin column chromatography (750 × 1000 mm). The separation by resin-based column was executed as following: extract solution (pH 10) was loaded onto the column. After adsorption, the column was washed with deionised water to remove the polar impurities, and then eluted with 100% MeOH to obtain the crude alkaloid extracts. The combined extract was dried by rotator evaporation at 40 °C. This residue was further separated by silica gel column chromatography using a system of CH_2_Cl_2_/MeOH (100%→0%) gradient, 80% CH_2_Cl_2_/MeOH, saturated with ammonia, to give eight subfractions. After solvent removal sub-fraction 2 (242 mg) gave a single spot. The residue was a colourless amorphous solid (16.3 mg, compound **1**). Sub-fraction 3 (870 mg), a single spot after removal of the solvent, was recovered from methanol-acetone to give **2** (26 mg). Sub-fraction 7 (218 mg) was chromatographed by MPLC on an ODS column using MeOH–H_2_O (5:1) with addition of 0.1% trifluoroacetic acid (TFA) to afford **3** (8.5 mg). From sub-fraction 4 (115 mg), compounds **4** (3.6 mg, t*_R_* = 18.6 min) and **5** (3.6 mg, t*_R_* = 21.1 min) were purified by semi-preparative HPLC (using a gradient solvent system of MeOH-0.1% TFA (25:75 → 85:15; flow rate 5 mL/min) over 90 min; UV detection at 210 nm; YMC Pak ODS-A column (20 × 250 mm, 5 μm particle size)]. Compound **6** (5.5 mg; t*_R_* = 28.6 min) was isolated from fraction 5 (212 mg) by semi-preparative HPLC (using a gradient solvent system of MeOH-0.1% TFA (20:80 → 80:20; flow rate 5 mL/min) over 90 min; UV detection at 210 nm; YMC Pak ODS-A column (20 × 250 mm, 5 μm particle size)].

### 3.4. Lycosquarosine A *(**1**)*

White amophous powder; [α]25 *D* = −6.54 (*c* 0.05, CHCl_3_); UV (CHCl_3_) *λ*_max_ (log ε): 255 (3.50) nm; IR *ν*_max_ (KBr): 3397, 1685, 1630, 1452, 865 cm^−1^; HR-ESI-MS *m/z* 320.1868 [M+H]^+^ (calcd for C_18_H_25_NO_4_, 320.1885); ^1^H- (CDCl_3_) and ^13^C-NMR (CDCl_3_) data are listed in Table 1.

### 3.5. In Vitro Cholinesterase Inhibitory Activity Assay

The AChE and BChE inhibitory activities were measured using the spectrophotometric method developed by Ellman *et al.* with a slight modification. ACh and BCh were used as the substrates to detect the inhibition of AChE and BChE, respectively. The reaction mixture contained sodium phosphate buffer (pH 8.0, 140 μL); tested sample solution (20 μL); and either AChE or BChE solution (20 μL), which were mixed and incubated for 15 min at room temperature. All tested samples and the positive control (berberine) were dissolved in 10% analytical grade dimethyl sulfoxide. The reactions were started with the addition of DTNB (10 μL) and either ACh or BCh (10 μL), respectively. The hydrolysis of ACh or BCh was monitored by following the formation of the yellow 5-thio-2-nitrobenzoate anion at 412 nm for 15 min, which resulted from the reaction of DTNB with thiocholine, released by the enzymatic hydrolysis of either ACh or BCh, respectively. All reactions were performed in triplicate and recorded in 96-well microplates, using a VERSA max instrument (Molecular Devices, Sunnyvale, CA, USA). Percent inhibition was calculated from the expression (1 − S/E) × 100, where E and S were the respective enzyme activities without and with the tested sample, respectively. The ChE inhibitory activity of each sample was expressed in terms of the IC_50_ value (μM required to inhibit the hydrolysis of the substrate, ACh or BCh, by 50%), as calculated from the log-dose inhibition curve [19].

## 4. Conclusions

Six lycopodium alkaloids, including a new natural product, lycosquarosine A, were isolated from the club moss *H. squarrosa*. This is the first report on the alkaloid constituents of *H. squarrosa* from Vietnam and the potential cholinesterase inhibitory activity of these compounds might suggest new sources of anti-Alzheimer disease agents.

## Supplementary Materials

Supplementary materials can be accessed at: http://www.mdpi.com/1420-3049/19/11/19172/s1.

## Supplementary Files

Supplementary File 1
