# Supplementary material for: Anti-Cholinesterase Activity of Lycopodium Alkaloids from Vietnamese Huperzia squarrosa (Forst.) Trevis
Source: Molecules. 2014 Nov 19;19(11):19172–9. doi: 10.3390/molecules191119172 (PMC6271335; doi:10.3390/molecules191119172)

Supplementary Information

Figure S1. ^{1}H-NMR spectrum of compound **1**.

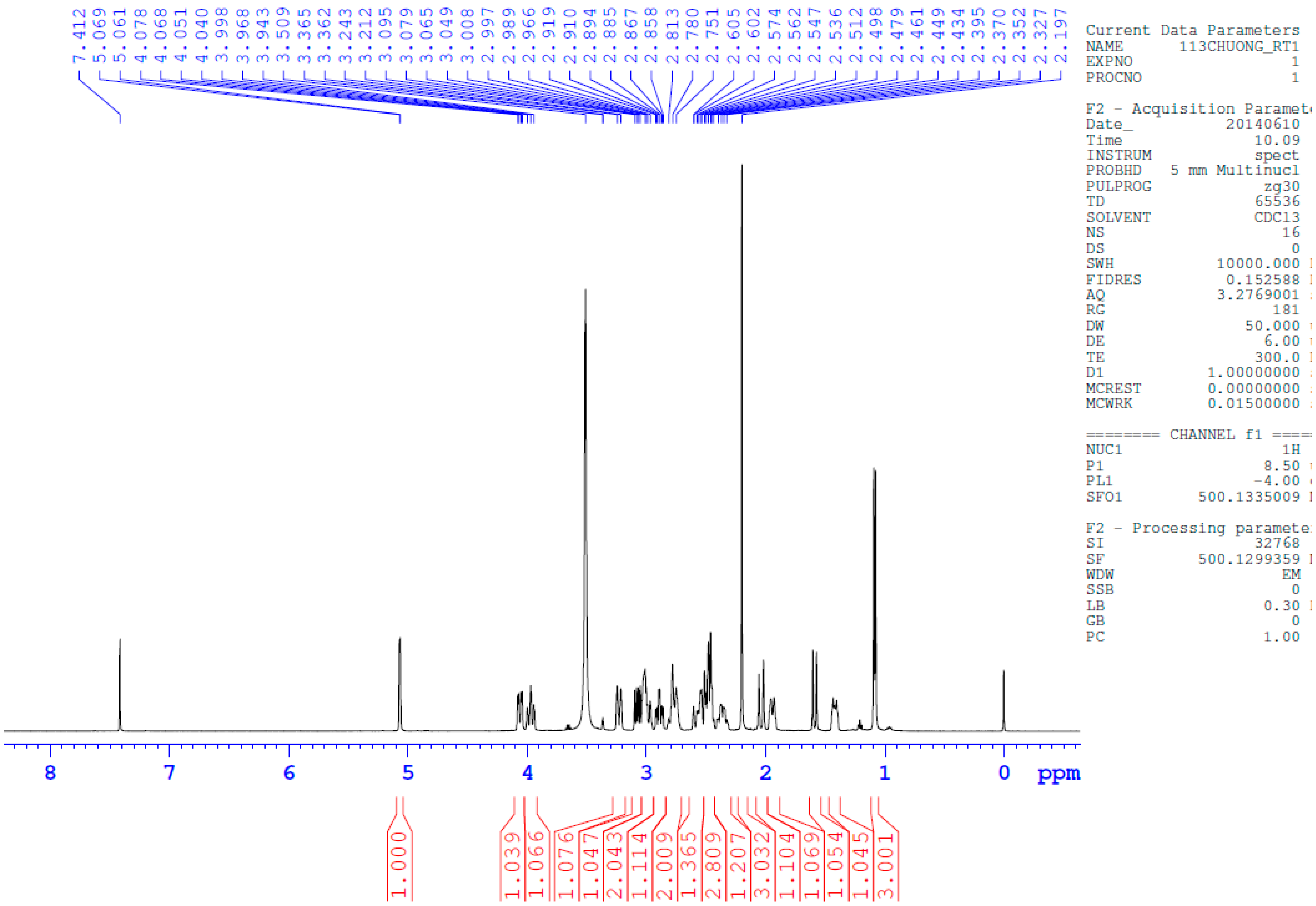

Figure S2. ^{13}C-NMR spectrum of compound **1**.

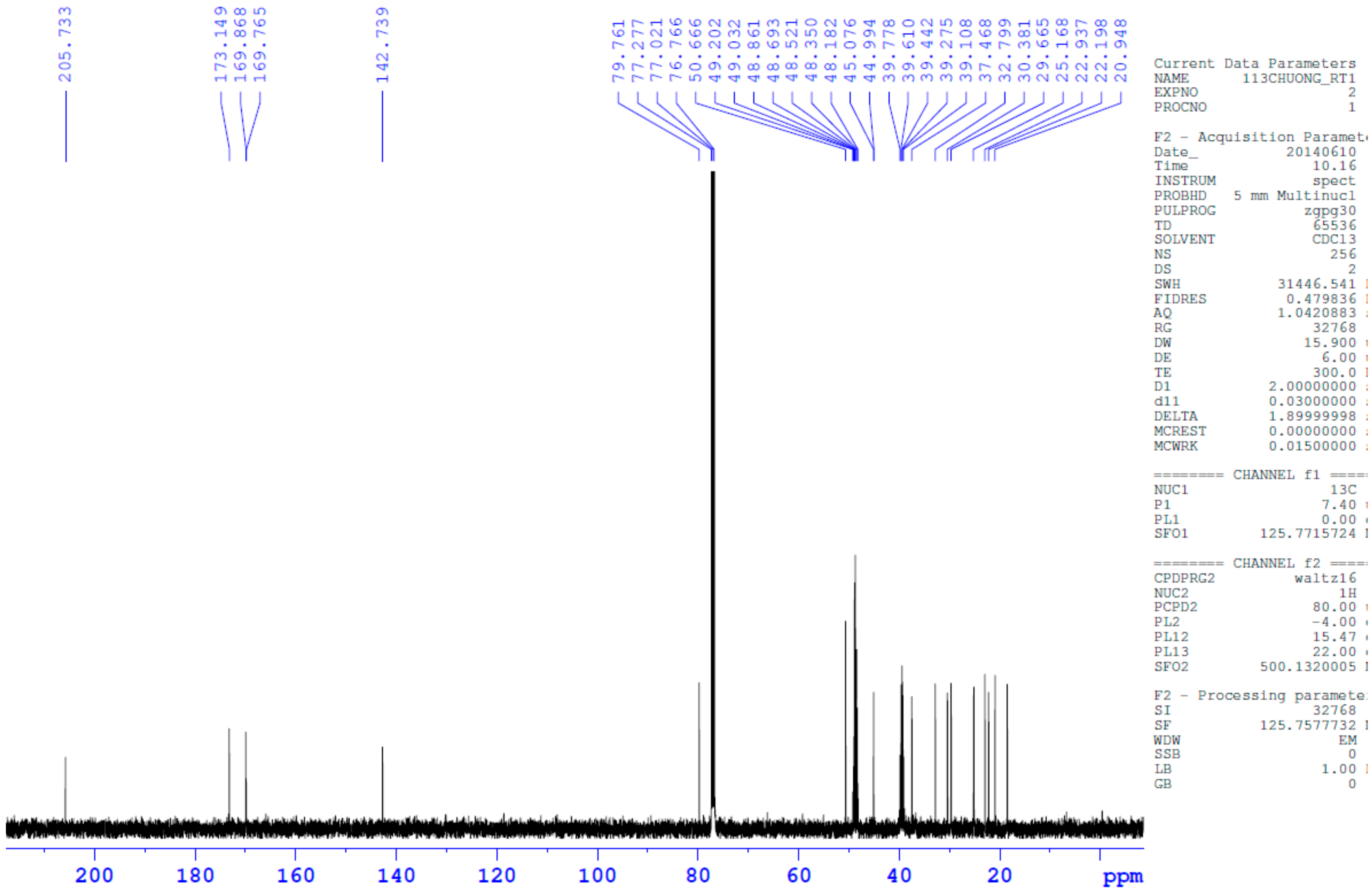

Figure S3. HMQC spectrum of compound **1**.

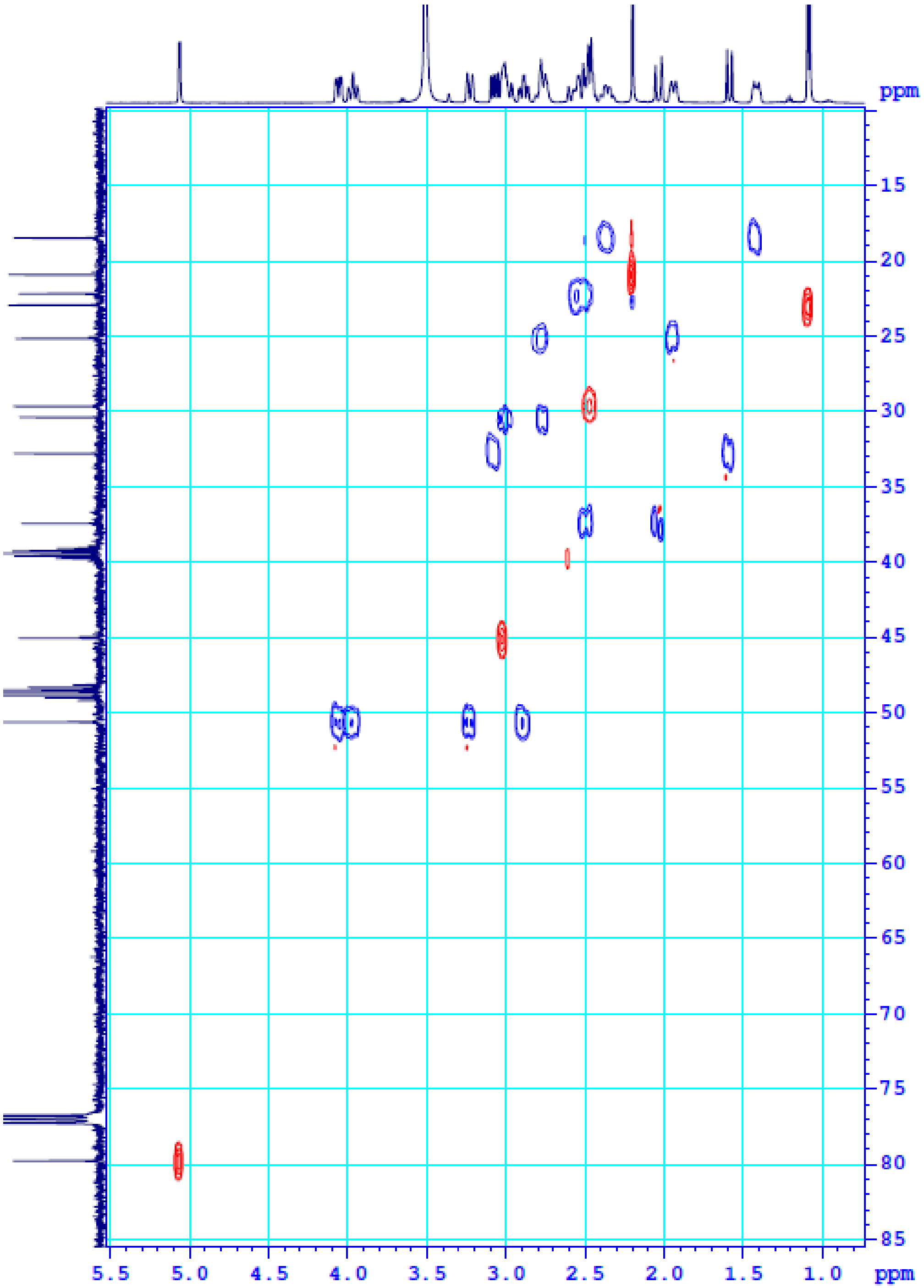

Figure S4. HMBC spectrum of compound **1**.

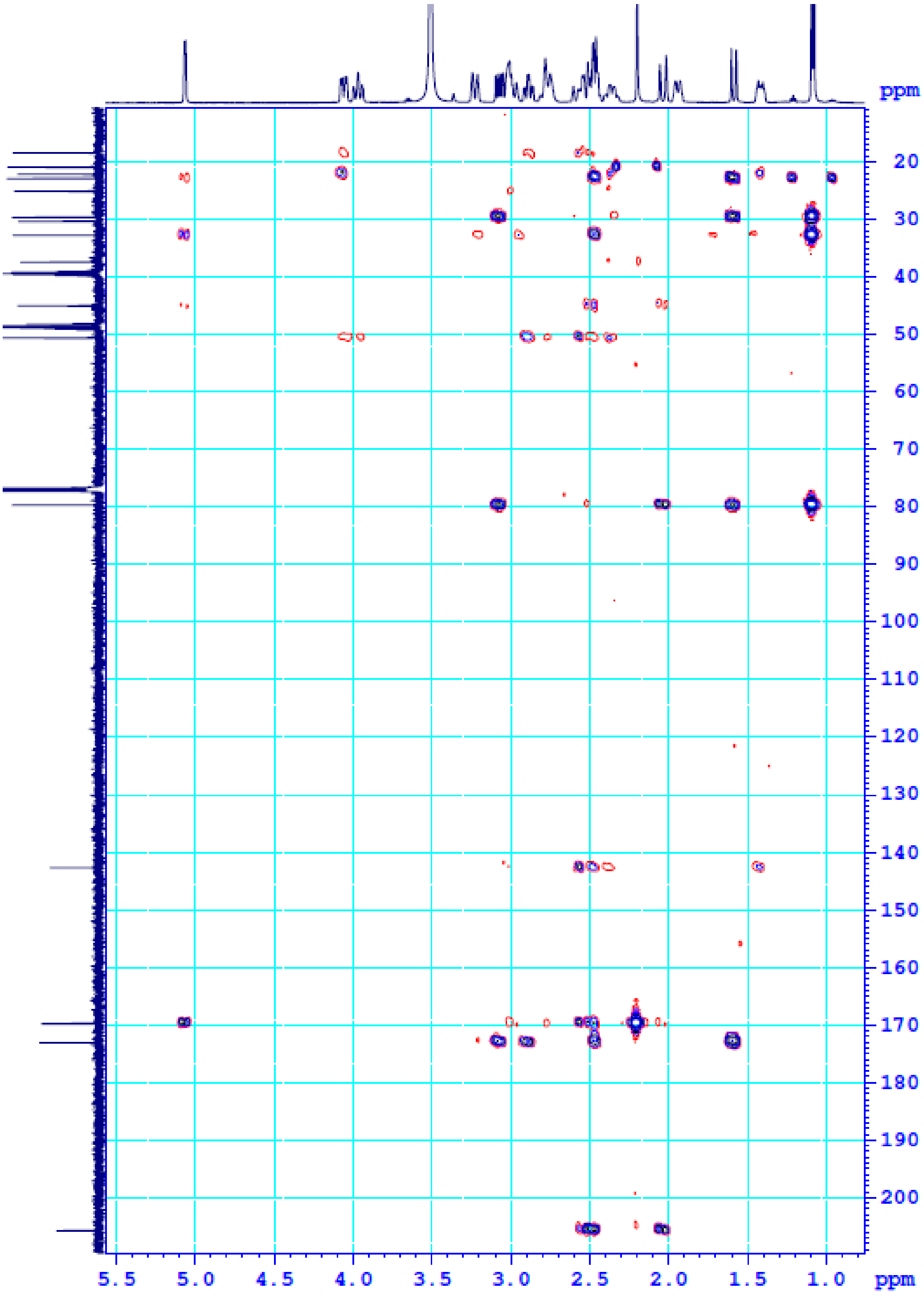

Figure S5. COSY spectrum of compound **1**.

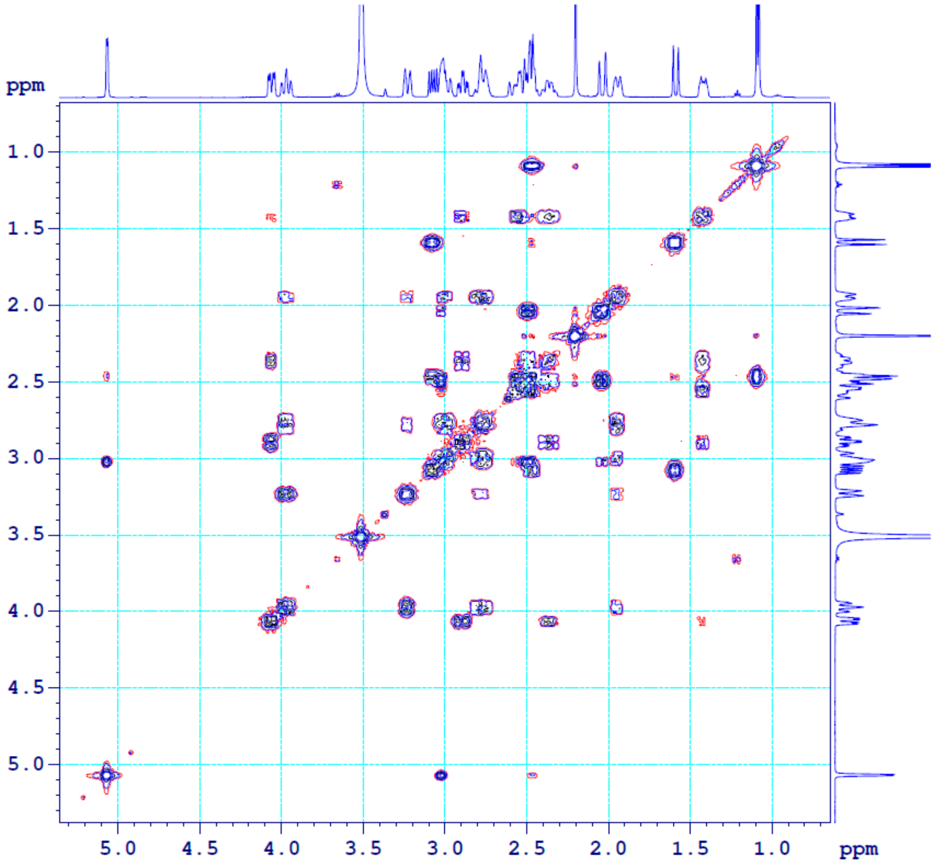

Figure S6. ROESY spectrum of compound **1**.

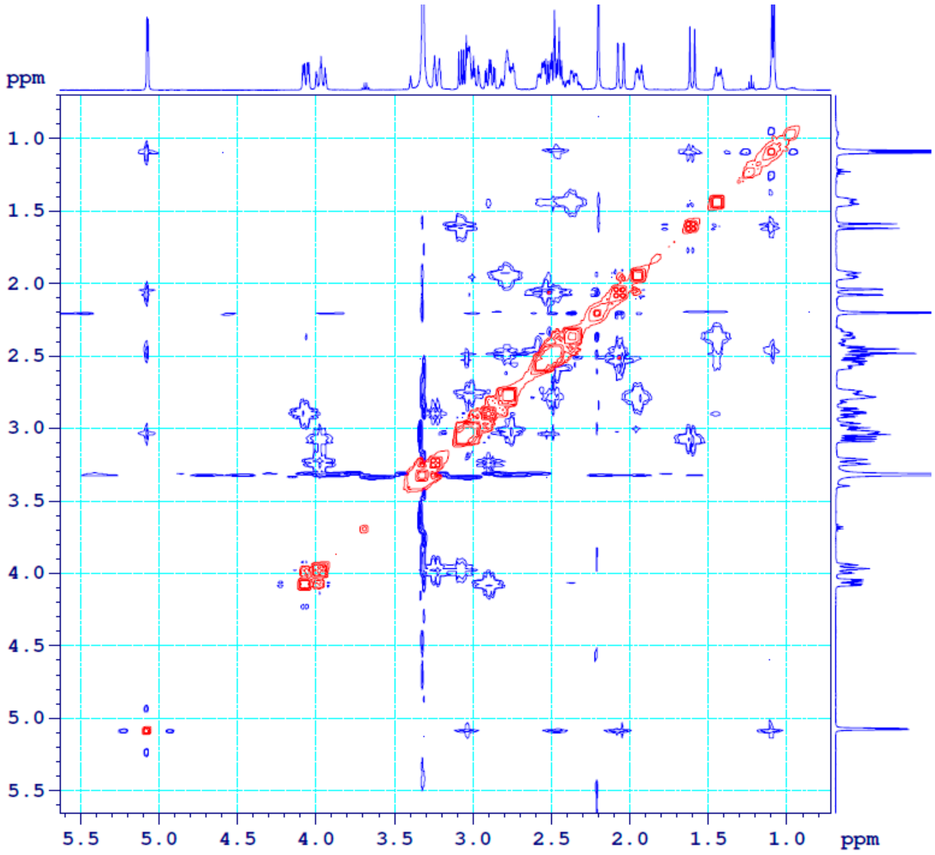

Figure S7. HRESIMS of compound **1**.

Display Report

Analysis Info

Analysis Name	D:\Data\datadmm 2012\RT6_1-B,6_01_7257.d	Acquisition Date	4/8/2013 8:48:43 PM
Method	thaytai.m	Operator	Mai
Sample Name	RT6	Instrument	micrOTOF-Q 10187
Comment			

Acquisition Parameter

Source Type	ESI	Ion Polarity	Positive	Set Nebulizer	1.2 Bar
Focus	Not active	Set Capillary	4500 V	Set Dry Heater	200 °C
Scan Begin	50 m/z	Set End Plate Offset	-500 V	Set Dry Gas	8.0 l/min
Scan End	2000 m/z	Set Collision Cell RF	150.0 Vpp	Set Divert Valve	Source

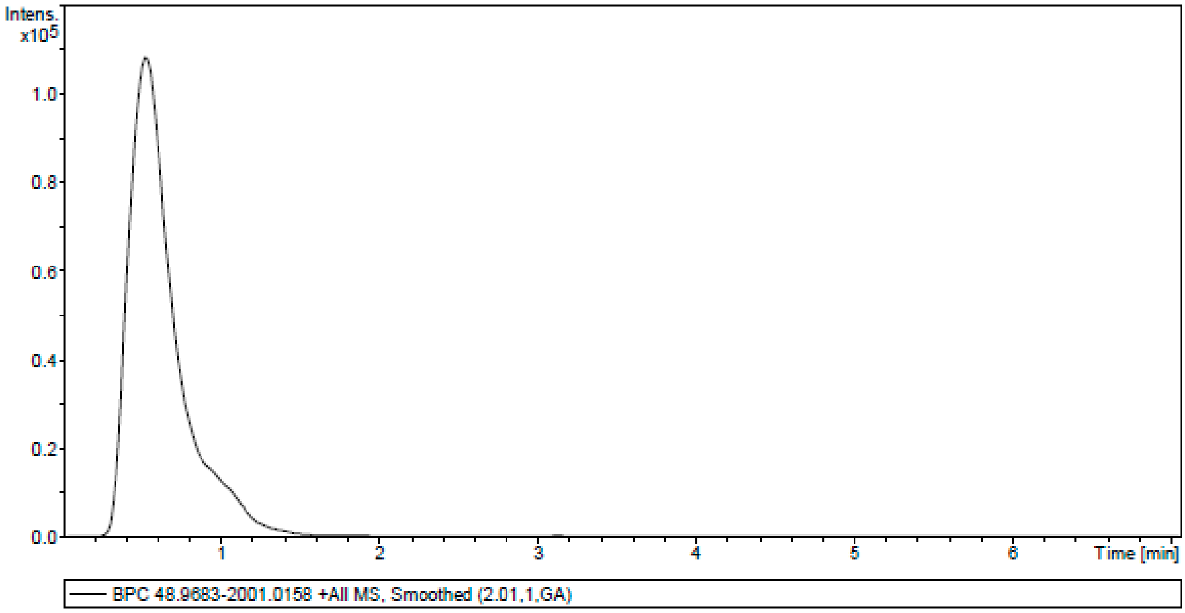

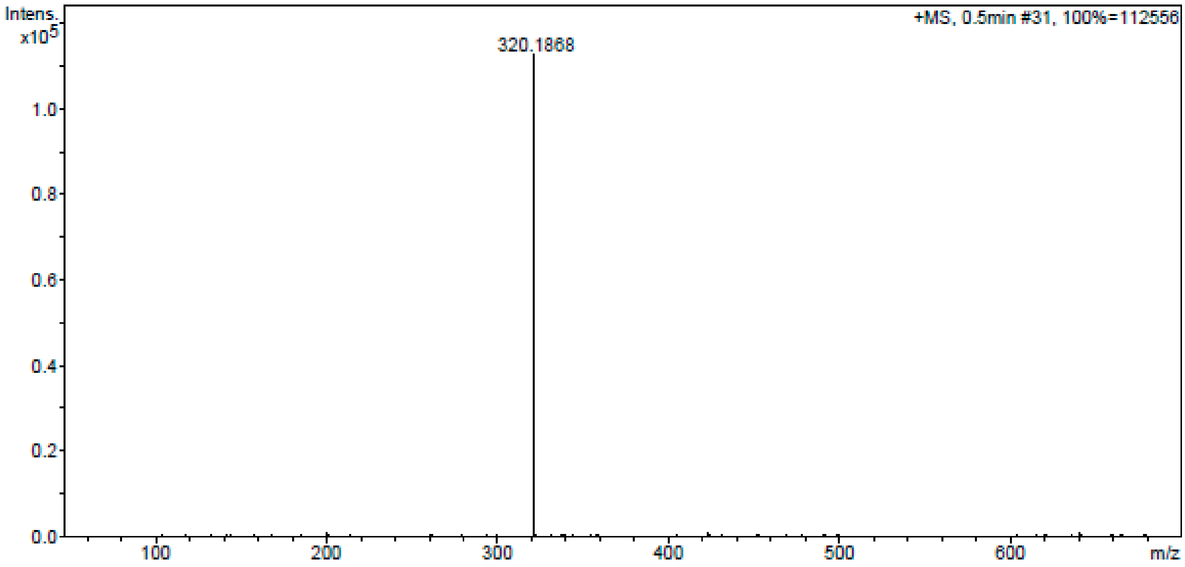

Supplement: Supplementary File 1 [file molecules-19-19172-s001.pdf]
